# A Multicenter Retrospective Analysis on the Etiology of Bradycardia in COVID-19 Patients

**DOI:** 10.7759/cureus.21294

**Published:** 2022-01-16

**Authors:** Chukwuemeka Umeh, Curren Giberson, Sabina Kumar, Mahendra Aseri, Pranav Barve

**Affiliations:** 1 Internal Medicine, Hemet Global Medical Center, Hemet, USA; 2 Data Engineering and Business Intelligence, Hemet Global Medical Center, Hemet, USA

**Keywords:** mortality, beta-blocker, steroid, remdesivir, bradycardia, sars-cov2, covid-19

## Abstract

Introduction

Bradycardia has been reported in the setting of SARS-CoV2 (COVID-19) and appears to be an important cardiac manifestation with an association of mortality. However, the etiology of bradycardia in COVID-19 remains unclear. Therefore, this study aims to retrospectively investigate the potential causes of bradycardia in COVID-19 patients.

Method

The multicenter retrospective analysis consisted of 1,116 COVID-19 positive patients from March 2020 to March 2021. Bradycardia and severe bradycardia were defined as a sustained heart rate of <60 BPM and <50 BPM, respectively, on two separate occasions, a minimum of four hours apart during the hospitalization. End-of-life bradycardia was excluded from the study. Data were retrieved using a structured query language (SQL) program through the EMR, and data were analyzed using IBM SPSS 27.0 (IBM Corp., Armonk, NY). Logistic regression was used to study the bradycardic event and its association with remdesivir, beta-blockers, or steroids use during the patient's hospital stay.

Result

In the multivariate analysis, bradycardia was significantly associated with length of hospital stay (p<0.001), mortality (p=0.022), ventilator use (p=0.001), and steroid use (p=0.001). However, there was no significant association between bradycardia and remdesivir use (p=0.066) or beta-blocker use (p=0.789).

Conclusion

Our study showed that steroid use was protective against developing bradycardia in COVID-19 patients. Furthermore, remdesivir and the use of beta-blockers were not associated with bradycardia in COVID-19 patients. However, bradycardia was associated with both increased mortality and length of stay in the hospital. Therefore, future studies should focus on the mechanism of bradycardia in COVID-19 patients and the effect of bradycardia on patient outcomes.

## Introduction

Coronavirus disease 2019 (COVID-19), caused by severe acute respiratory syndrome coronavirus 2 (SARS-CoV-2), originated in Wuhan, China, in December 2019 and has since spread to nearly every country on the planet. As of November 2021, there have been 258 million cases and 5.16 million deaths worldwide. In the United States alone, there have been 47.9 million confirmed cases and 770,890 deaths [1].

SARS-CoV-2, like many viruses, must bind to a cell surface protein to facilitate entry into host cells. It utilizes the receptor-binding domain (RBD) of its spike (S) protein to bind to human angiotensin-converting enzyme-2 (ACE2) [2], a protein present on nearly all human tissues but particularly abundant in the heart, kidneys, gastrointestinal tract, and respiratory system [3]. There is high expression of the ACE2 gene in nasal epithelial cells, possibly contributing to the high infectivity and rapid spread of SARS-CoV-2 [4]. Expression of the RAS is a complex and highly regulated process that pathogens can manipulate. Some viruses, such as SARS-CoV-2, induce transcription of type 1 interferon (IFN-a), a pro-inflammatory cytokine produced by human respiratory epithelial cells, indirectly enhancing expression of ACE2 and facilitating viral entry into host cells [5].

The symptoms of SARS-CoV-2 infection highly correlate with the organs that have the highest ACE2 expression. Infection of the respiratory and gastrointestinal epithelium produces common symptoms such as dyspnea, cough, and diarrhea. However, a less-publicized implication of COVID-19 is cardiac damage. Patients without cardiovascular comorbidities who become infected with SARS-CoV-2 have an increased incidence of arrhythmias, cardiomyopathies, acute coronary syndromes, coagulopathies, and myocarditis [6], while patients with preexisting cardiovascular disease have increased mortality [7]. While the exact pathophysiology is unclear, SARS-CoV-2 arrhythmias are thought to arise from electrolyte abnormalities, acidosis, hypoxemia, and inflammation resulting from the massive release of cytokines, also known as a cytokine storm [8].

One proposed mechanism for the bradyarrhythmia seen in COVID-19 infection is the correlation between the cytokine storm produced by severe SARS-CoV-2 infection and elevated interleukin-6 (IL-6) [9]. IL-6 has been shown to directly affect the sinoatrial node, causing increased vagal tone and decreased heart rate [6]. In addition, medications used in treating COVID-19 are also a possible cause of bradycardia in COVID-19 patients [10,11]. The rate of bradyarrhythmia among all COVID-19 patients in Wuhan, China, has been reported as high as 16.7% [12], but the incidence increases significantly among hospitalized patients with severe COVID-19 [9,13]. The most common bradyarrhythmias among hospitalized COVID-19 patients were sinus bradycardia and first-degree heart block [14]. While tachyarrhythmias are more common in COVID-19 patients, bradyarrhythmia has been associated with higher mortality and more severe disease [15]. Therefore, this study aims to retrospectively investigate the potential causes of bradycardia in COVID-19 patients.

## Materials and methods

We performed a multicenter retrospective analysis, which included two Southern California hospitals, on patients with a COVID-19 diagnosis verified by PCR between March 2020 and March 2021. A total of 1,116 patients were identified. Relevant deidentified patient data were extracted using a structured query language (SQL) program from the electronic medical record, which included: age, gender, race, comorbidities, laboratory results on admission, date of admission, date of discharge, medications they received while on admission, heart rate, and disposition at discharge. Bradycardia and severe bradycardia were defined as a sustained heart rate <60 beats per minute and <50 beats per minute, respectively, on two separate occasions, a minimum of four hours apart during the hospitalization [16]. End-of-life bradycardia was excluded from the study.

We performed a univariate analysis of the independent variables, including patients' age, gender, ethnicity, marital status, comorbidities, the medication patients received while in the hospital, and laboratory results, using means and percentages. Furthermore, we performed a bivariate analysis of the relationship between bradycardia and different study variables using chi-square and t-test, with a P-value of 0.05 considered significant. Finally, we performed a backward selection logistic regression to study the relationship between bradycardia, remdesivir, and steroid use. The effect was expressed as an odds ratio with a 95% confidence interval. Statistical analysis was done using IBM SPSS version 27. The WIRB-Copernicus Group (WCG) institutional review board (IRB) approved the study, and the study's IRB approval number is 13410516.

## Results

We had 1,116 patients in the study, with a mean age of 66 years and a range of 19 to 101 years. The mean hospital length of stay was nine days and ranged from 0 to 64 days (Table 1). Forty-nine percent of the patients were female, 82% were white, and 26% expired. Forty-five percent of the patients received remdesivir, and 66% received steroids (dexamethasone or methylprednisolone; Table 1).

**Table 1:** Descriptive statistics of all patients in the study

	Mean	Standard deviation
Age	65.52	17.512
Body mass index	30.786	8.9036
Length of hospital stay	9.03	8.199
	Frequency	Percent
Gender		
Female	541	48.5%
Male	575	51.5%
Race		
White	900	81.6%
Black	80	7.2%
Others	125	11.2%
Expired		
No	821	73.6%
Yes	295	26.4%
Ventilator use		
No	909	81.5
Yes	207	18.5%
ICU admission		
No	878	78.7
Yes	238	21.3%
Remdesivir		
No	609	54.6%
Yes	507	45.4%
Steroid use (dexamethasone or methylprednisolone)		
No	381	34.1%
Yes	735	65.9%
Diabetes		
No	617	55.3%
Yes	499	44.7%
Hypertension		
No	442	39.6%
Yes	674	60.4%
Chronic kidney disease		
No	889	79.7%
Yes	227	30.3%
Acute kidney injury		
No	822	73.7%
Yes	294	26.3%
Coronary artery disease		
No	910	81.5%
Yes	206	18.5%
Congestive heart failure		
No	922	82.6%
Yes	194	17.4%
Chronic obstructive pulmonary disease		
No	959	85.9%
Yes	157	14.1%
Beta-blocker use		
No	770	69%
Yes	346	31%
Pulse <50 beats per minute		
No	1039	93.1
Yes	77	6.9
Pulse <60 beats per minute		
No	740	66.3%
Yes	376	33.7%

In the bivariate analysis, mortality (p=0.001), ventilator use (p<0.001), intensive care unit admission (p<0.001), diabetes (p=0.002), hypertension (p=0.02), remdesivir use (p<0.001), steroid use (p<0.001), and calcium channel blocker use (p=0.003) were significantly associated with bradycardia (Table 2).

**Table 2:** Bivariate analysis of the relationship between categorical variables and bradycardia

Variable	Bradycardia		P-value
	No	Yes	
Gender			
Male	64.5%	35.5%	0.193
Female	68.2%	31.8%	
Race			
White	66.6%	33.4%	0.413
Black	70.0%	30.0%	
Others	61.6%	38.4%	
Expired			
Yes	58.3%	41.7%	0.001
No	69.2%	30.8%	
Ventilator use			
Yes	44.0%	56.0%	<0.001
No	71.4%	28.6%	
Intensive care unit admission			
Yes	49.6%	50.4%	<0.001
No	70.8%	29.2%	
Diabetes			
Yes	61.5%	38.5%	0.002
No	70.2%	29.8%	
Hypertension			
Yes	63.6%	36.4%	0.02
No	70.4%	29.6%	
Chronic kidney disease			
Yes	69.6%	30.4%	0.239
No	65.5%	34.5%	
Acute kidney injury			
Yes	61.9%	38.1%	0.063
No	67.9%	32.1%	
Congestive heart failure			
Yes	64.9%	35.1%	0.659
No	66.6%	33.4%	
Chronic obstructive pulmonary disease			
Yes	70.7%	29.3%	0.209
No	65.6%	34.4%	
Coronary artery disease			
Yes	65.5%	34.5%	0.795
No	66.5%	33.5%	
Remdesivir use			
Yes	57.0%	43.0%	<0.001
No	74.1%	25.9%	
Steroid use			
Yes	59.2%	40.8%	<0.001
No	80.1%	19.9%	
Beta-blocker use			
Yes	65.0%	35.0%	0.544
No	66.9%	33.1%	
Calcium channel blockers use			
Yes	58.9%	41.1%	0.003
No	68.7%	31.3%	
Statin			
Yes	65.2%	34.8%	0.479
No	67.2%	32.8%	

In the backward selection logistic regression multivariate analysis, bradycardia was significantly associated with length of hospital stay (p<0.001), mortality (p=0.022), ventilator use (p=0.001), and steroid use (p=0.001). However, there was no significant association between bradycardia and remdesivir use (p=0.066) or beta-blocker use (p=0.789) (Table 3).

**Table 3:** Multivariate analysis of the relationship between bradycardia and different variables

	B	S.E.	Wald	df	P-value	Odds ratio	95% CI for odds ratio	
							Lower	Upper
Length of hospital stay	0.069	0.011	42.748	1	0.000	1.071	1.049	1.094
Age	0.008	0.004	3.158	1	0.076	1.008	0.999	1.016
Expired	0.470	0.205	5.239	1	0.022	1.600	1.070	2.394
Ventilator use	−0.755	0.233	10.480	1	0.001	0.470	0.298	0.742
Steroid use	−0.582	0.168	12.018	1	0.001	0.559	0.402	0.776
Diabetes	−0.264	0.139	3.605	1	0.058	0.768	0.585	1.009
Beta-blocker use	0.041	0.154	0.072	1	0.789	1.042	0.770	1.410
Remdesivir use	−0.278	0.151	3.378	1	0.066	0.758	0.563	1.019

## Discussion

Our analysis showed that 34% of COVID-19 patients had bradycardia, while 7% had severe bradycardia during their hospital stay. The cause of bradycardia in our study is unclear as bradycardia was not associated with atrioventricular nodal blocking agents such as beta-blockers. Prior studies have also noted that bradycardia in COVID-19 patients was not associated with hypoxia, myocardial ischemia, or medications that induce bradycardia [16,17]. Therefore, it is possible that the bradycardia in our study was caused by a direct pathogenic effect of COVID-19 on the myocardium or conduction system or that the COVID-19 infection worsened preexisting myocardial or conduction system conditions in the patients [18]. In addition, bradycardia was associated with an increased length of hospital stay and mortality in our study. The association of bradycardia with increased mortality has been reported in previous studies; however, the mechanism remains unclear [16,17,19]. One possible explanation is that the inflammatory cytokine storm in COVID-19 both causes bradycardia by its effect on pacemaker cells and also causes increased mortality [16,17,19].

Our study showed that the use of dexamethasone or methylprednisolone was protective against bradycardia, which means that patients on these steroids were significantly less likely to have bradycardia. Contrary to our finding, steroids have been reported to cause bradycardia in non-COVID-19 patients, especially after using high or pulse-dose steroids [20-22]. We had expected that steroid use would result in bradycardia in COVID-19 patients, but the reverse was the case. The mechanism through which steroids provide a protective effect against bradycardia is unclear. A possible explanation is that bradycardia in COVID-19 is caused by damage to the myocardium and conduction system from inflammatory system activation and cytokine storm [19]. In this scenario, steroids inhibit the severe inflammatory process of COVID-19 and reduce bradycardia in COVID-19 patients [23]. Another possible explanation is that corticosteroids act on beta-adrenergic receptors in the heart, leading to positive inotropic and chronotropic effects [23,24]. Thus, the increased heart rate from steroid use may counteract the bradycardia caused by COVID-19 infection.

Furthermore, contrary to prior studies, our study did not show any association between remdesivir use and bradycardia. Some authors have reported bradycardia in COVID-19 patients on remdesivir, which happens through an unclear mechanism [25-28]. It has been postulated that remdesivir may cause bradycardia by slowing the sinoatrial node's automaticity through its active metabolite, a nucleotide triphosphate derivative with a similarity to adenosine triphosphate (ATP) [26,27]. However, no randomized controlled trial has reported increased bradycardia in patients on remdesivir. Thus, the bradycardia seen in observational studies and case reports might have been due to confounders.

The strengths of our study include a multicenter study with a large sample size (>1000 patients) that relied on clinical, laboratory, and outcome data collection. Additionally, we adjusted for possible confounders that would have affected bradycardia, such as the use of beta-blockers. However, our study was limited by its retrospective cohort design. Our study initially included all patients with a documented COVID-19 infection from two southern California hospitals. In addition, the data collection was dependent on the results entered into the electronic medical record, which could cause some discrepancies. Despite our careful analysis, it is not possible to fully account for all potential confounders that might affect the outcome of our study.

## Conclusions

Our study demonstrated that dexamethasone or methylprednisolone use was protective against developing bradycardia in COVID-19 patients. Furthermore, remdesivir and the use of beta-blockers were not associated with bradycardia in COVID-19 patients. However, bradycardia was associated with increased mortality and an increased length of hospital stay. Therefore, in the future, studies should focus on the mechanism of bradycardia in COVID-19 patients and the effect of bradycardia on patient outcomes.
